# Central role for neurally dysregulated IL-17A in dynamic networks of systemic and local inflammation in combat casualties

**DOI:** 10.1038/s41598-023-33623-z

**Published:** 2023-04-24

**Authors:** Ruben Zamora, Jonathan A. Forsberg, Ashti M. Shah, Desiree Unselt, Scott Grey, Felipe A. Lisboa, Timothy R. Billiar, Seth A. Schobel, Benjamin K. Potter, Eric A. Elster, Yoram Vodovotz

**Affiliations:** 1 Department of Surgery, University of Pittsburgh, W944 Starzl Biomedical Sciences Tower, 200 Lothrop St., Pittsburgh, PA 15213 USA; 2 Department of Surgery, Uniformed Services University of Health Sciences and Walter Reed National Military Medical Center, Bethesda, MD 20814 USA; 3 Center for Inflammation and Regeneration Modeling, McGowan Institute for Regenerative Medicine, Pittsburgh, PA 15219 USA; 4 Pittsburgh Liver Research Center, University of Pittsburgh, Pittsburgh, PA 15213 USA; 5 Surgical Critical Care Initiative (SC2i), Uniformed Services University of Health Sciences, Bethesda, MD 20814 USA; 6 The Henry M Jackson Foundation for the Advancement of Military Medicine, Bethesda, MD 20817 USA

**Keywords:** Systems biology, Biomarkers, Medical research

## Abstract

Dynamic Network Analysis (DyNA) and Dynamic Hypergraphs (DyHyp) were used to define protein-level inflammatory networks at the local (wound effluent) and systemic circulation (serum) levels from 140 active-duty, injured service members (59 with TBI and 81 non-TBI). Interleukin (IL)-17A was the only biomarker elevated significantly in both serum and effluent in TBI vs. non-TBI casualties, and the mediator with the most DyNA connections in TBI wounds. DyNA combining serum and effluent data to define cross-compartment correlations suggested that IL-17A bridges local and systemic circulation at late time points. DyHyp suggested that systemic IL-17A upregulation in TBI patients was associated with tumor necrosis factor-α, while IL-17A downregulation in non-TBI patients was associated with interferon-γ. Correlation analysis suggested differential upregulation of pathogenic Th17 cells, non-pathogenic Th17 cells, and memory/effector T cells. This was associated with reduced procalcitonin in both effluent and serum of TBI patients, in support of an antibacterial effect of Th17 cells in TBI patients. Dysregulation of Th17 responses following TBI may drive cross-compartment inflammation following combat injury, counteracting wound infection at the cost of elevated systemic inflammation.

## Introduction

Twenty years of combat in Iraq and Afghanistan have left many service members with combat-related trauma, often combined with traumatic brain injury (TBI). This “signature injury” of war^1^ is a pathophysiology driven, in part, by dysregulated systemic inflammation. Despite an increase in injury severity and extensive systemic inflammation, all mainly due to blast injuries, combat-related injury survival has improved substantially due to upgraded combat armor and modern developments in combat casualty care. Early studies have suggested that measurement of the systemic and local response to injury using inflammatory biomarkers may predict combat wound healing outcomes^2^ and introduce personalized medicine into combat casualty care^3^.

The extensive body of literature regarding TBI and trauma (reviewed in^4,5^) demonstrates that the resulting inflammatory processes are complex, individual-specific, injury-specific and associated with morbidity and mortality^6^. The cellular and molecular mediators of the innate and adaptive immune system do not function in isolation, but rather through dynamic interactions to either stimulate or suppress inflammation^7^. Our understanding of how inflammation and immune dysregulation affect clinical outcomes in settings such as TBI has advanced, in part, through mathematical and computational modeling^1,6,8^.

We have sought to address the complexity of post-injury inflammation using data-driven and mechanistic computational modeling^6,9–13^. Using soluble and cellular circulating multi-omics in human trauma, these studies have defined novel inflammatory biomarkers^2,13–20^, endotypes^13,21^, and regulatory programs^13,20,22^. A key, unanswered question centers around how systemic inflammation impacts injury-induced local inflammatory responses in wounds on systemic inflammation and vice versa, i.e., if inflammation in one compartment modulates the response in the other^23^. We have begun to address this question in experimental paradigms of trauma/hemorrhage via multiple computational approaches including hypergraph analysis, a graph-theory approach to modeling multi-compartment trends in inflammation^24^.

Here, we sought to leverage and expand this computational methodology to provide insights into the interplay between local and systemic inflammation following combat injury in humans. We hypothesized that trauma patients with TBI have a more complex dynamic, cross-compartment inflammatory network pattern than trauma patients without TBI and sought to identify specific mediator connections that may yield mechanistic insights into those differences.

## Materials and methods

### Patient and sample selection

Samples used in this study were obtained from combat-wounded active-duty service members who sustained high-energy extremity injuries and were enrolled prospectively between 2008 and 2012 under a protocol approved by Walter Reed National Military Medical Center IRB (IRBNet #352354). The study was conducted as a prospective study in accordance with all relevant guidelines and regulations and was approved by the Institutional Review Board from the participating institution. Written informed consent was obtained for all patients during enrollment. Samples of peripheral venous blood (serum) and wound effluent were collected at the operating room before each surgical debridement (approximately every 3 days). Wound effluent was collected from only the most extensive wound from each patient. Injury-specific and demographic data, as well as 23 serum and wound effluent inflammation biomarkers assessed during each debridement (see below), were collected from 59 combat casualties with poly trauma that included TBI ([TBI], age: 24.5 ± 0.8 y.o.) and 81 casualties with injuries that did not include TBI ([non-TBI], age: 24.4 ± 0.6 y.o). TBI was confirmed by clinical evaluation of patient mental status after injury and confirmed with CT scans as appropriate.

### Analysis of inflammatory mediators

Inflammatory mediators in both the serum and effluent samples were assayed in batch using a multiplex analysis platform (Luminex™ 100 System, Luminex, Austin, TX). Samples were thawed, filtered using a 0.65 μm filter (Millipore, Billerica, MA), and tested using human cytokine Luminex kits (MPXHCYTO-60K-06 and MPXHCYTO-60K-17, Millipore Billerica, MA, USA) for the level of: Eotaxin (CCL11), Granulocyte–Macrophage Colony-Stimulating Factor (GM-CSF), Interferon (IFN)-α2, IFN-γ, Interleukin (IL)-1α, IL-1β, IL-2, IL-3, IL-4, IL-5, IL-6, IL-7, IL-8, IL-10, IL-12p40, IL-12p70, IL-13, IL-15, IL-17A, Interferon-γ-Inducible Protein 10 (IP-10/CXCL10), Monocyte Chemoattractant Protein-1 (MCP-1/CCL2), Macrophage Inflammatory Protein-1α (MIP-1α, CCL3), and Tumor Necrosis Factor-α (TNF-α). Results were analyzed using the software BeadView (Upstate V1.0.4.23259, Millipore, Billerica, MA) and concentrations expressed in pg/ml and represented as mean ± SEM.

### Statistical and computational analyses

*Two-way analysis of variance (ANOVA)* was carried out to compare changes in inflammatory mediators over time (debridement intervals) and TBI status using *SigmaPlot* (Systat Software, San Jose, CA) as indicated (significance set at P < 0.05).

*Dynamic network analysis (DyNA)*^14,25–27^ was carried out to define inflammatory network interconnectivity as a function of both patient sub-group and time, quantified as debridement interval, wherein each debridement takes place approximately every 3 days. Networks were created over four consecutive debridement intervals (n1-n2, n2-n3, n3-n4, n4-n5) using MATLAB® software as described previously^14,25–27^. Connections, defined as the number of trajectories of serum inflammatory mediators that move in parallel (black edges) or in anti-parallel (red edges) fashion across time intervals, were created if the Pearson correlation coefficient between any two nodes (inflammatory mediators) at the same time-interval was greater or equal to a threshold value of 0.95, as indicated. The network complexity for each time-interval was calculated using the following formula:

$$\sum \begin{array}{c}\frac{{N}_{1}+{N}_{2}+\dots +{N}_{n}}{n-1}\\ \end{array}$$

where N represents the number of connections for each mediator and n is the total number of mediators analyzed.

To show the number of connections for each mediator, we employed MetaboAnalyst (https://www.metaboanalyst.ca), a web-based tool suite developed for comprehensive metabolomic data analysis that also supports a wide array of functions for statistical, functional, as well as data visualization tasks^28,29^.

*Shannon entropy* was utilized as another form of quantification of network complexity. First, we calculated the probability of mediator connection for each mediator in each debridement interval (= number of connections/total number of possible connections) and used an online calculator (https://calculator1.net/maths/shannon-entropy-calculator) to compute the Shannon entropy for each network.

*Spearman’s correlation analysis* was carried out to measure the strength of association of IL-17A with GM-CSF (suggesting the presence of pathogenic Th17 cells), IL-10 (suggesting the presence of non-pathogenic Th17 cells), and TNF-α (suggesting the presence of memory/effector T cells) using a modified version of a MATLAB® -based toolbox described previously^17,30^.

*Dynamic hypergraph (DyHyp) analysis* (a spatial extension of static hypergraph modeling^31^) was carried out to map inflammatory mediators that were significantly increasing or significantly decreasing (Pearson’s correlation coefficient, |r|> 0.95) over three consecutive debridement intervals. The independent variable for calculating Pearson’s correlation coefficient was time (i.e., if the dynamic interval included debridement intervals n1, n2, n3 then the independent variables were 0, 3 and 6) and the dependent variable was mean Luminex™ quantification of a specific inflammatory mediator within either serum or wound effluent (reported as pg/mL). Pearson’s correlation coefficient was calculated for each mediator in the serum and effluent independently across 3 dynamic intervals: n1-n3, n2-n4, and n3-n5.

A hypergraph is a type of nodal graph model in which edges have the special property of connecting more than two nodes^24,32–35^. Here, we define edges as inflammatory mediators and nodes to be tissue compartments (either serum or wound effluent). If an edge is statistically correlated with itself over three consecutive time periods independently in multiple tissues, an edge (mediator) is drawn connecting the two or more nodes in which the mediator was significantly correlated with time. If two or more mediators are correlated independently over time in the same compartment, or same set of compartments, then only one edge is drawn connecting the respective set of nodes; the given edge is labeled with the names of all the mediators that are statistically significant in that set of compartments.

The dynamic hypergraphs described above have two quantitative properties: edge distribution and edge weight. Edge distribution is a count of the number of significant edges that are present in serum, wound effluent, or both serum and effluent. Study of such a distribution over time intervals provides insight into the spatial shifts in inflammation over time. Edge weight represents the strength (Pearson’s correlation) of increase or decrease in a cytokine value over a dynamic time interval. Here, we show only those mediators that have a weight, or Pearson’s correlation, greater than 0.95 or less than − 0.95.

### Conference presentation

Part of this work was presented at the 2016 Military Health System Research Symposium (MHSRS). Abstract # MHSRS-16-1473 - Precision Medicine as well as the 2022 International Conference on Complex Acute Illness.

## Results

### Patient population characteristics

Patients (n = 140, age: 24.5 ± 0.5 y.o) were segregated into two groups based on the presence or absence of overt, clinically diagnosed TBI. Of these, 59 TBI individuals (age: 24.5 ± 0.8 y.o.; 98.3% males) were compared with 81 non-TBI casualties (24.4 ± 0.6 y.o; 100% males). Injury-specific and demographic data for TBI vs. non-TBI individuals with both serum and effluent samples available for analysis are shown in Table 1. TBI individuals had a higher frequency of multiple extremity wounds (P < 0.05). However, no statistically significant differences were found in heterotopic ossification (P = 0.284) or wound healing (healed vs. failed, P = 0.505) between TBI and non-TBI patients (Table 1).

**Table 1:** Clinical characteristics of trauma patients with extremity wounds (TBI vs. non-TBI, analyzed by Chi-square).

Descriptor	Traumatic brain injury		P-value
	No, n = 77	Yes, n = 53	
Gender = Male (%)	77 (100.0)	52 (98.1)	0.850
Nicotine use = Yes (%)	35 (45.5)	24 (45.3)	0.873
Wound type (%)			
Transfemoral amputation	9 (11.7)	14 (26.4)	
Transtibial amputation	19 (24.7)	15 (28.3)	
Transhumeral amputation	0 (0.0)	1 (1.9)	
Transradial amputation	0 (0.0)	1 (1.9)	
Foot amputation	1 (1.3)	0 (0.0)	
Open fracture	30 (39.0)	12 (22.6)	
Closed fracture	1 (1.3)	0 (0.0)	
Knee disarticulation	1 (1.3)	3 (5.7)	
Shoulder disarticulation	0 (0.0)	1 (1.9)	
Soft tissue injury	16 (20.8)	6 (11.3)	
Number of wounds (%)			
Single	26 (33.8)	4 (7.5)	**0.001**
Multiple	51 (19.5)	49 (26.4)	**0.001**
Wound appearance (%)			
All debridement surgeries	240	154	
Gross purulence and necrotic tissue	13 (5.4)	12 (7.8)	0.464
Some necrotic tissue but no purulence	93 (38.8)	52 (33.8)	0.371
Some purulence but no necrotic tissue	4 (1.7)	4 (2.6)	0.785
No purulence or necrotic tissue	130 (54.2)	85 (55.2)	0.923
HO wound (%)			0.284
No	42 (54.5)	22 (41.5)	
Yes	35 (45.5)	30 (56.6)	
Unknown	0 (0.0)	1 (1.9)	
Final outcome = Healed (%)	67 (87.0)	43 (81.1)	0.505

Significant values are in bold.

### Wound-localized and circulating levels of IL-17A are higher in combat casualties with trauma + TBI vs. trauma patients without TBI

**Figure 1:**
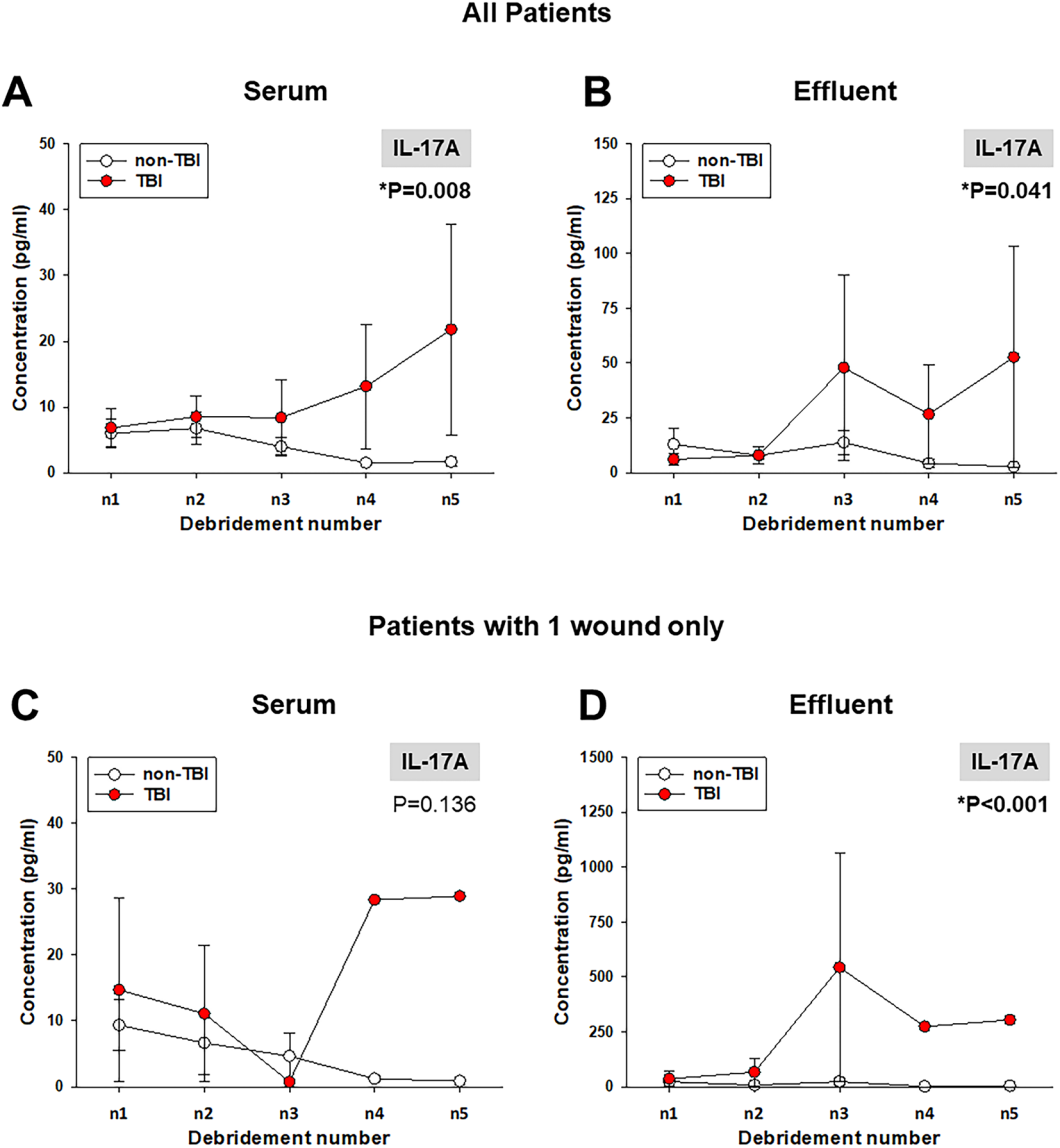
Levels of IL-17A in serum and wound effluent samples from TBI and non-TBI trauma patients. Inflammatory mediators in serum and wound effluent samples of trauma patients taken during five consecutive debridements were measured by Luminex™ as described in “Materials and methods”. Comparison of levels of IL-17A in both serum (**A**) and wound effluent (**B**) samples shows significant elevation in TBI vs. non-TBI patients. Comparison of levels of IL-17A in serum (**C**) and wound effluent (**D**) samples of patients with 1 wound only as described in *Results* (*P < 0.05, analyzed by Two-Way ANOVA as described in “Materials and methods”).

We assessed the post-injury time courses of 23 immune mediators in both serum and wound effluent samples. Analysis by Two-Way ANOVA demonstrated significant differences between the chronological expression of multiple serum and effluent inflammatory mediators between combat casualties with or without TBI (Suppl. Fig. S1 [serum] and Suppl. Fig. S2 [wound effluent]), including IFN-α2, IFN-γ, IL-12p40, IL-12p70, IL-17A, and IL-2 in serum; and IL-1α, IL-17A, and IL-5 in the wound effluent. Notably, IL-17A was the only significantly elevated biomarker in both serum (Fig. 1A) and wound effluent (Fig. 1B) of casualties with TBI as compared to casualties without TBI.

As TBI patients were more likely than non-TBI patients to have multiple wounds (Table 1), it is possible that the elevated circulating levels of IL-17A might be associated with a more extensive injury in the TBI patients when compared to the non-TBI patients. To address this possibility, we compared the levels of IL-17A in both serum and effluent of a smaller subgroup of TBI and non-TBI patients with a single wound. As shown in Fig. 1C,D, levels of IL-17A were higher in serum (Fig. 1C) and significantly higher in wound effluent of patients with TBI (Fig. 1D) as compared to non-TBI patients, suggesting that the observed upregulation of IL-17A in the larger cohort was likely dependent on the presence of TBI rather than on the number of wounds.

### Single-compartment dynamic network analysis demonstrates higher network complexity in serum vs. wound effluent as well as higher network complexity and IL-17A connectivity in casualties with TBI

**Figure 2:**
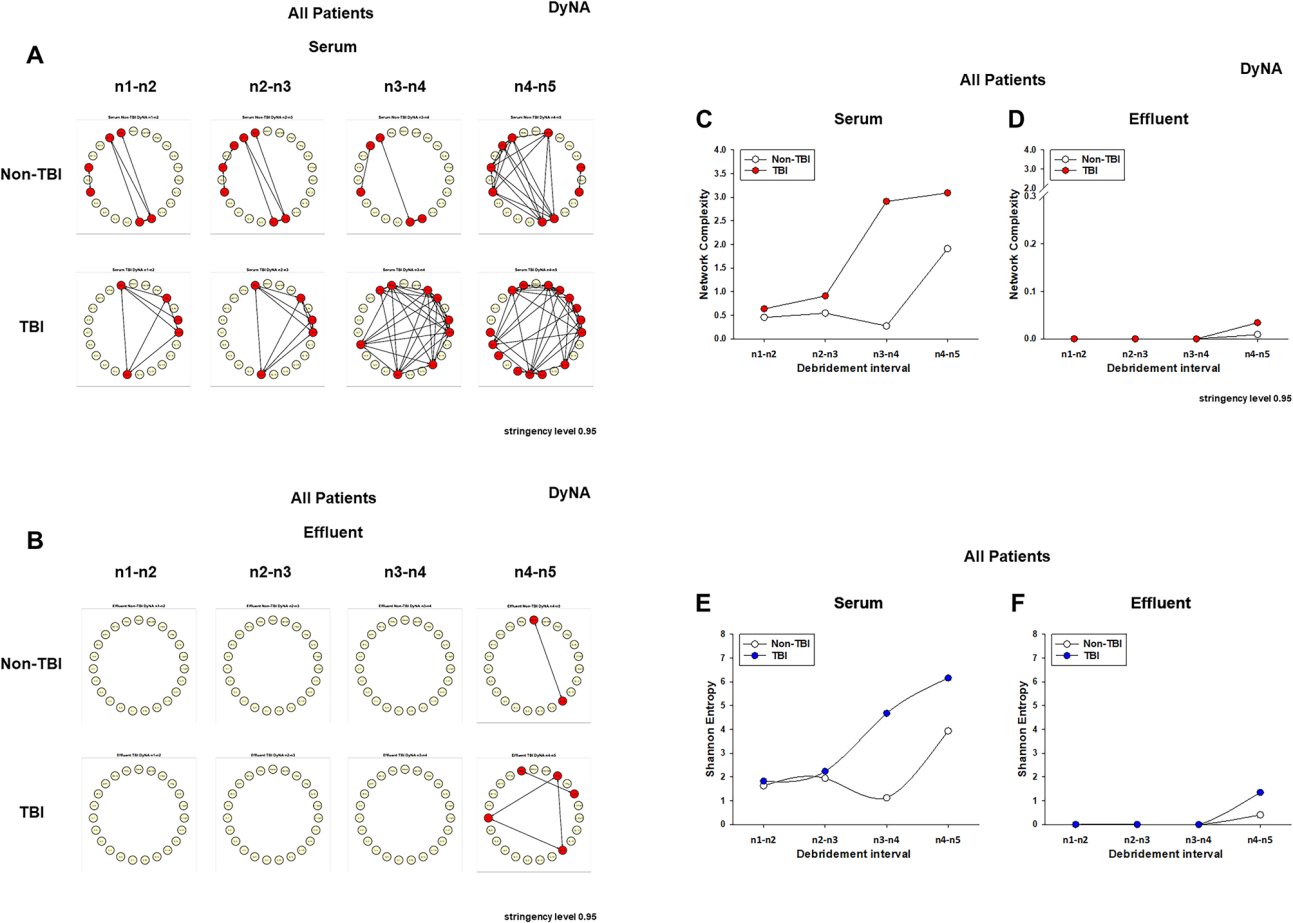
Differential dynamic inflammatory networks in TBI vs. non-TBI trauma patients. Circulating inflammatory mediators in serum samples from 59 TBI and 81 non-TBI patients were measured, and DyNA (stringency level = 0.95) was performed during each of four debridement intervals (n1-n2, n2-n3, n3-n4, n4-n5) using MATLAB® software as described in “Materials and methods”. (**A**,**B**) Show an overview of all the networks and mediator connections in both patient subgroups in (**A**) serum and (**B**) effluent (the closed circles represent mediators with at least one connection to another mediator, while open circles represent mediators that had no connections to other mediators as determined by DyNA). (**C** (serum),** D** (effluent)) Shows the network complexity for each patient subgroup during each of the four debridement intervals calculated as described in “Materials and methods”. (**E** (serum), **F** (effluent)) Shows the Shannon Entropy for each network calculated as described in “Materials and methods”.

**Figure 3:**
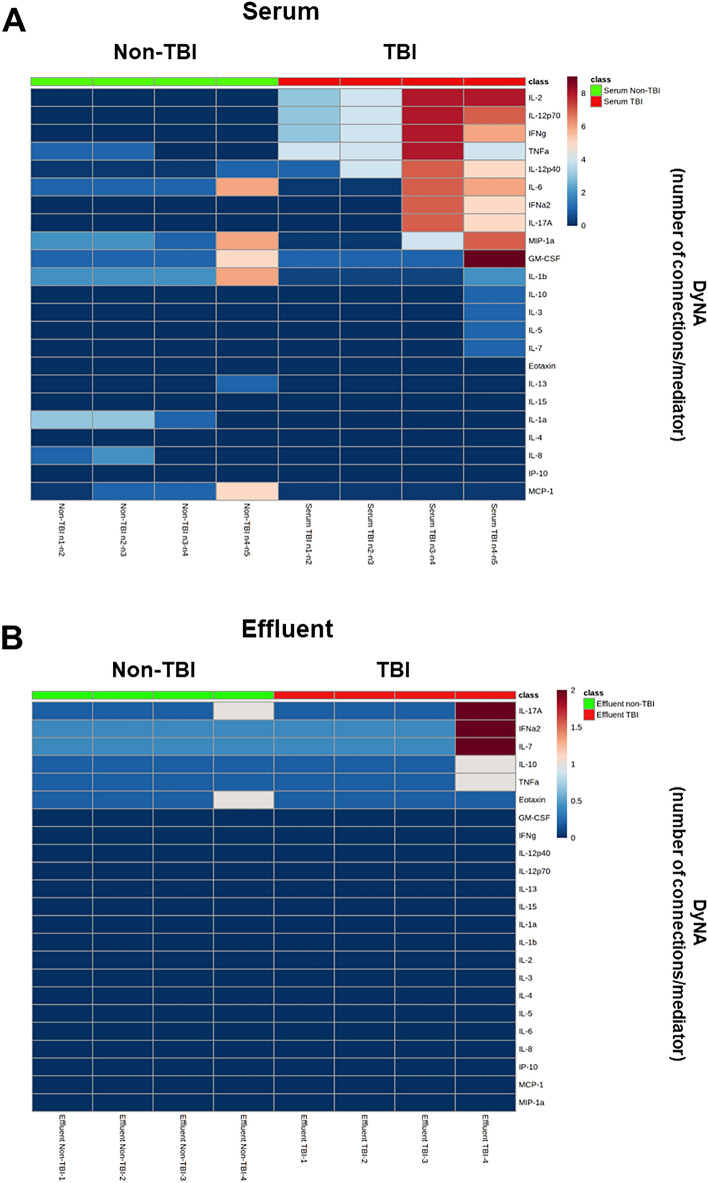
Dynamic Network Analysis (DyNA) of circulating inflammatory mediators TBI vs. non-TBI trauma patients. Circulating inflammatory mediators in serum and effluent samples from 59 TBI and 81 non-TBI patients were measured, and DyNA (stringency level = 0.95) was performed during each of four debridement intervals (n1-n2, n2-n3, n3-n4, n4-n5) using MATLAB® software as described for Fig. 2. Figure represents the specific number of connections/mediator and debridement interval in TBI vs. non-TBI patients shown as a heatmap (serum [**A**] and effluent [**B**]) using MetaboAnalyst 5.0 (https://www.metaboanalyst.ca) and calculated as described in “Materials and methods”.

We next hypothesized that the presence of TBI would be reflected in dynamic inflammatory networks. As an initial test of this hypothesis, DyNA^14,26,36^ was employed to define and compare the interconnections among inflammatory mediators separately in wound effluent and serum of TBI vs. non-TBI patients over four defined debridement intervals (n1-n2, n2-n3, n3-n4, n4-n5). DyNA showed differential dynamic inflammation networks in serum from TBI as compared to non-TBI patients (Fig. 2A). In contrast, inflammatory network connectivity was absent or minimal at most defined debridement intervals in the wound effluent of trauma patients with or without TBI (Fig. 2B). However, at the final debridement interval (n4-n5), wound effluent network connectivity was slightly higher in patients with TBI (Fig. 2B). These features are quantified in Fig. 2C,D. Furthermore, assessment of the degree of interaction among inflammatory mediators using Shannon entropy (Fig. 2E,F) mirrored the results shown in Fig. 2C,D. In both serum (Fig. 3A) and effluent (Fig. 3B), trauma patients with TBI exhibited higher mediator connectivity for several inflammatory mediators including IL-17A, IFNα2, IFNγ, IL-12p40, IL-12p70, and IL-2 (see Suppl. Table S1 for number of connections/mediator). We focused on IL-17A connectivity because, as noted above, IL-17A was the only biomarker significantly elevated in both serum (Fig. 1A) and wound effluent (Fig. 1B) of casualties with TBI as compared to casualties without TBI.

Based on the data available, we also segregated all patients according to the type of wound injury (Blast vs. Gunshot wound [GSW]) as shown in Table 2. None of the TBI patients had GSW, so we could not generate inflammatory networks specific for that type of injury. However, DyNA in patients who sustained injuries due to Blast only resulted in essentially the same inflammatory networks and network complexity for both the serum and the effluent of both non-TBI and TBI patients (Suppl. Fig. S3). This suggests that the differences observed in the inflammatory networks shown in Fig. 2A–D (all patients) were due to the presence of TBI rather than the injury mechanism.

**Table 2:** Number of patients segregated according to the type of wound injury (Blast vs. Gunshot wound [GSW]).

	Wound injury	Serum	Effluent
Non-TBI	GSW	18	17
	Blast	59	53
TBI	GSW	0	0
	Blast	53	50

### Correlation analyses suggest a differential profile of IL-17A-producing cell subsets in wound vs. systemic circulation of combat casualties

Interleukin-17A can be produced by different cell types, including Th17 cells, innate lymphoid cells, γδ T cells, and both CD4^+^ and CD8^+^ effector/memory T cells^37–39^. Two sub-populations of Th17 cells haven been identified: pathogenic Th17 cells, which are characterized by the co-expression of IL-17A and GM-CSF and are implicated in driving pathological inflammatory processes, and a reciprocal, non-inflammatory Th17 cell subset that co-expresses IL-17A and IL-10^40^. Furthermore, CD4^+^/CD8^+^ effector/memory T cells co-express IL-17A and TNF-α^41,42^. We have previously utilized Spearman rank correlation analysis of IL-17A vs. GM-CSF, IL-10, or TNF-α to infer the presence of these three IL-17A-producing cell subsets^17,21,24,43^, with positive and negative correlations being interpreted as increases or decreases, respectively, in these Th17 cell subsets.

**Figure 4:**
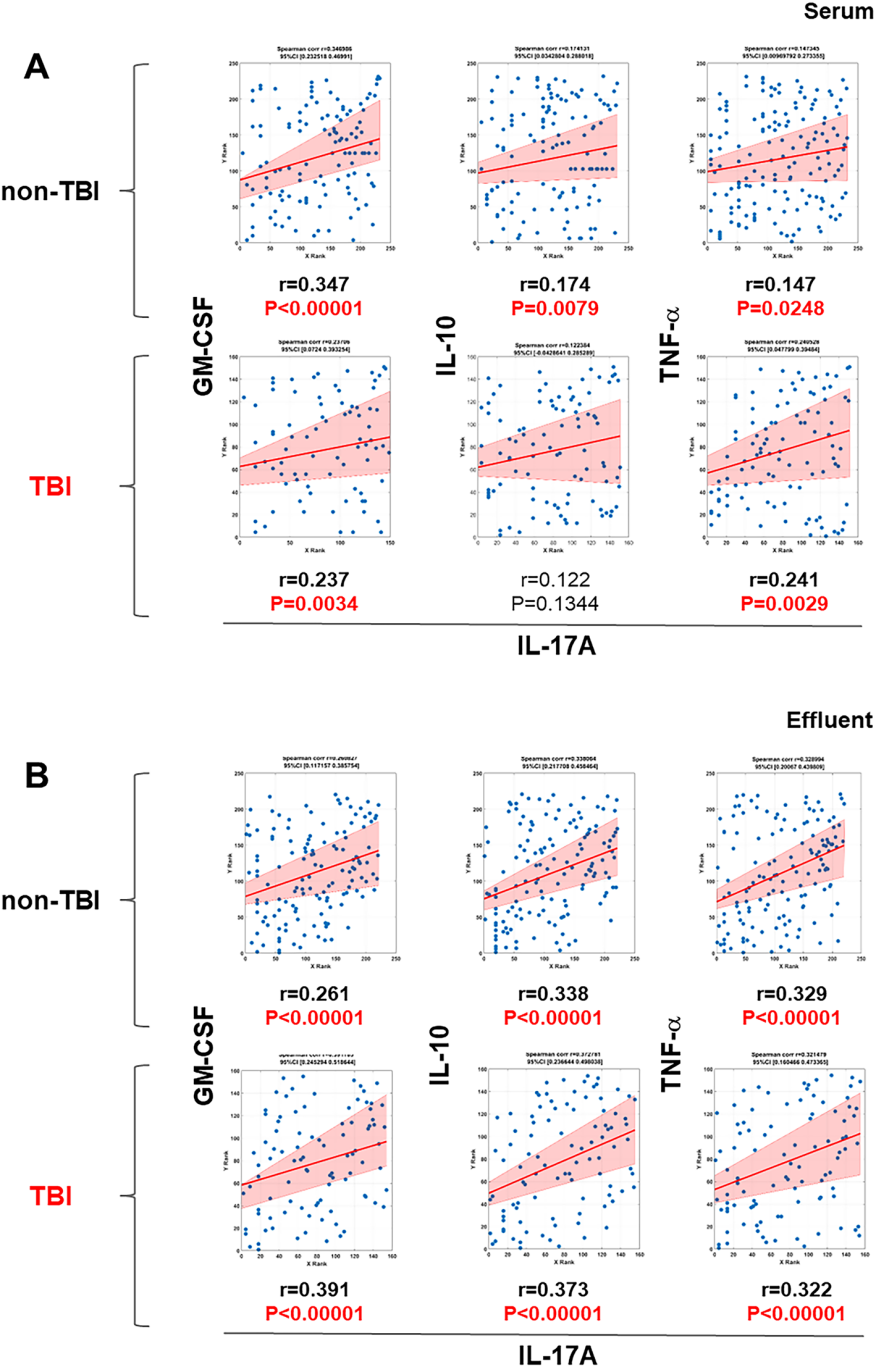
Correlation between levels of IL-17A/GM-CSF, IL-17A/IL-10 and IL-17A/TNFα in serum and wound effluent samples from TBI and non-TBI patients. Inflammatory mediators in serum and wound effluent samples of trauma patients taken during five consecutive debridements were measured by Luminex™ as described in “Materials and methods”. The plots show the Spearman’s correlations between levels of IL-17A/GM-CSF, IL-17A/IL-10 and IL-17A/TNFα in serum (**A**) or wound effluent samples (**B**) from TBI vs. non-TBI patients (the shaded area represents the 95% bootstrapped confidence interval).

We therefore carried out a similar analysis in serum samples of combat casualties with trauma ± TBI to infer the potential presence of these cells. As shown in Fig. 4A, the presence of both pathogenic (IL-17A/GM-CSF) and non-pathogenic (IL-17A/IL-10) Th17 cells was inferred in the non-TBI group as compared to only pathogenic Th17 cells in the TBI group. In contrast, we found a significant positive correlation for IL-17A and TNF-α, suggestive of memory/effector T cells, that was independent of the presence or absence of TBI. Similar correlation analysis in wound effluent samples resulted in significant positive correlations suggestive of pathogenic Th17 cells, non-pathogenic Th17 cells, and memory/effector T cells in all combat casualties regardless of the presence of TBI (Fig. 4B). This analysis suggests that the inclusion of TBI in the injury complex may be associated with an unbalanced circulating Th17 response that is skewed towards a pro-inflammatory phenotype.

### Cross-compartment dynamic network analysis suggests that TBI impacts the crosstalk of wound-localized and systemic inflammation following traumatic injury to counteract wound infection

**Figure 5:**
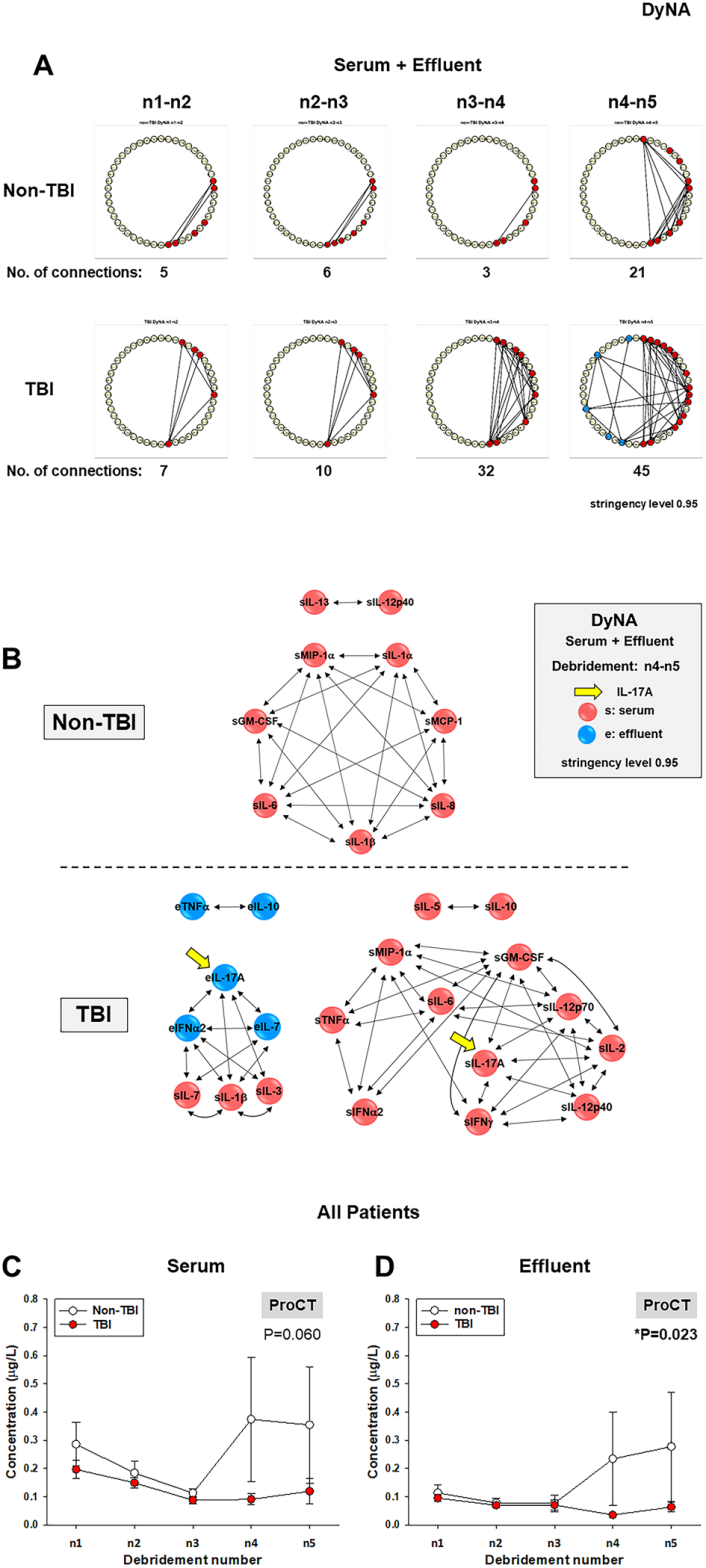
IL-17A connectivity in inflammatory networks and levels of ProCT in serum and wound effluent samples of TBI vs. non-TBI trauma patients. Circulating inflammatory mediators in serum and wound effluent samples combined from 59 TBI and 81 non-TBI patients were measured, and DyNA (stringency level = 0.95) was performed during each of four debridement intervals (n1-n2, n2-n3, n3-n4, n4-n5) using MATLAB® software as described in “Materials and methods” and Fig. 2*.* (**A**) Shows an overview of all the networks and mediator connections in both patient subgroups (the closed red and blue circles represent mediators with at least one connection to another mediator in serum and wound effluent, respectively, while open circles represent mediators that had no connections to other mediators as determined by DyNA) The total number of mediator connections for each debridement interval is shown below each network. (**B**) Shows the network complexity for each patient subgroup during the n4-n5 debridement interval calculated as described in “Materials and methods”. (**C**,**D**) Show levels of ProCT in serum and wound effluent samples from TBI and non-TBI trauma patients as a function of debridement number (*P < 0.05, analyzed by Two-Way ANOVA).

The overlap of IL-17A patterns between compartments in TBI patients led us to hypothesize that TBI impacts the crosstalk between wound-localized and systemic inflammatory responses. We therefore next sought to define the dynamic evolution of inflammatory networks in the wound effluent and systemic circulation in both patient sub-groups using DyNA as described above. This analysis showed differential dynamic inflammation networks and higher mediator connectivity in patients with TBI as compared to patients without TBI (Fig. 5A). We then focused on the IL-17A connectivity. At the final debridement interval (n4-n5), IL-17A had no network connections in patients without TBI, while both wound effluent and serum IL-17A were connected to multiple mediators in samples from patients with trauma and TBI. While effluent IL-17A was connected to both effluent (e) and serum mediators (s) (eIFNα2, eIL-7, sIL-1β and sIL-3), serum IL-17A was connected to serum mediators only (sGM-CSF, sIFNγ, sIL-2, sIL-12p40 and sIL12p70) (Fig. 5B). This analysis suggested that, in patients with TBI, circulating Th17 cells may traffic to wounds and drive a pro-inflammatory program. The DyNA results suggested that there was an increased efflux of pathogenic Th17 cells from the systemic circulation to the wounds of patients with trauma and TBI at later time points following injury, as evidenced by inferred connections from IL-17A to GM-CSF (Fig. 5B).

Various hypotheses may relate these IL-17A-related features to wound outcomes. One hypothesis is that pathogenic Th17 are involved in the recruitment of neutrophils to the wound to counteract wound infection. An alternative hypothesis is that these cells might be detrimental to wound closure by driving self-sustaining local inflammation. In support of the former hypothesis, levels of procalcitonin (ProCT, which is associated with bacterial infection/sepsis^44,45^) were lower in serum (Fig. 5C) and wound effluent of patients with trauma and TBI (Fig. 5D) as compared to non-TBI patients. Furthermore, as noted above, there were no statistically significant differences in heterotopic ossification or wound healing (healed vs. failed) between TBI and non-TBI patients, which contradicts the alternative hypothesis.

### Dynamic hypergraph analysis suggests that TBI disrupts inflammatory compartmentalization and promotes systemic inflammation

**Figure 6:**
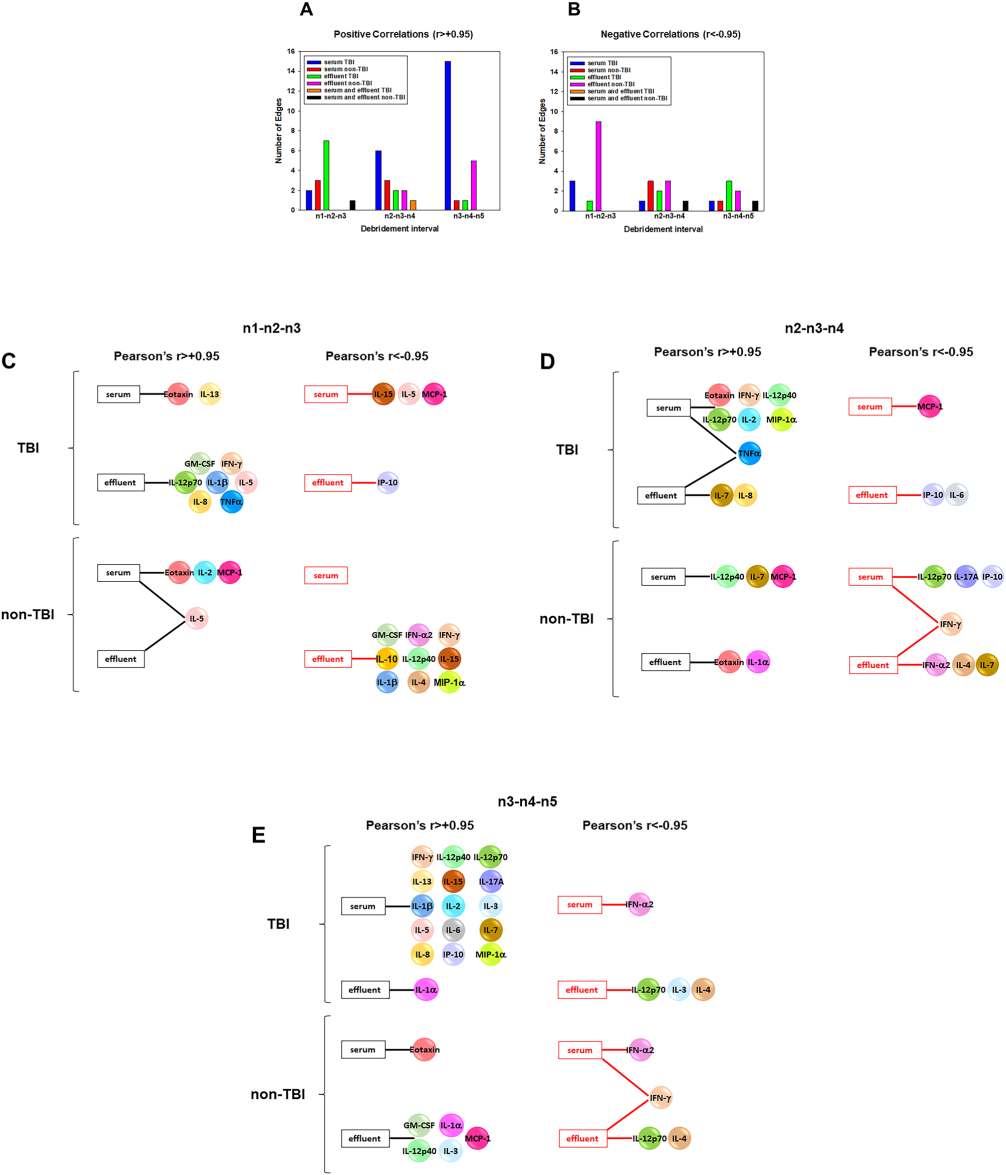
Dynamic hypergraphs propose the involvement of neural regulation in trans-compartmental inflammation. Dynamic hypergraphs indicate the statistically significant (P < 0.05; Pearson Correlation) growing or diminishing presence of an inflammatory mediator. (**A**) Demonstrates a shift in the number of upregulated inflammatory mediators from the effluent to the serum in TBI compared to non-TBI patients. (**B**) Depicts a greater number of downregulated inflammatory mediators in the effluent in non-TBI compared to TBI patients. (**C**–**E**) Depict significantly elevated/diminished cytokines over dynamic debridement intervals in both TBI (upper panels) and non-TBI (lower panels) patients.

We have previously employed static hypergraph analysis to gain insights into the spatiotemporal spread of inflammation across multiple tissues following experimental trauma/hemorrhage and resuscitation^24^. We expanded this methodology to gain an understanding of the processes that might regulate the interaction of wound-localized and systemic inflammation in combat casualties and have termed this novel method Dynamic Hypergraph (DyHyp) analysis. Edge distribution, a quantitative feature of dynamic hypergraphs, is a count of the number of edges that are found within a single or set of tissue compartments. In dynamic hypergraphs, a positive correlation across time for a given inflammatory mediators is inferred to reflect an inflammatory response that is strengthening over time. Likewise, a negative correlation across time for a given mediator suggests an inflammatory response that is attenuating over time. DyHyp analysis of edge distribution across debridement intervals suggested that the number of inflammatory mediators increased in the serum of patients with TBI during the five study events, defined by each debridement surgery. In contrast, the number of statistically significant inflammatory mediators in the wound effluent of TBI patients decreased over these same debridement intervals, suggesting that the majority of inflammatory networks are shifting from the local injury (effluent) assessed in this study to the systemic circulation over time (Fig. 6A). In non-TBI patients, the number of inflammatory mediators increasing in strength over time rose in the effluent samples but decreased in the serum (Fig. 6B). A Fisher’s Exact Test indicated that the positive edge distribution in the serum and effluent in TBI compared to non-TBI patients is independent of debridement interval (serum: p < 0.05; effluent p < 0.01). Based on this independence, we hypothesize that the number of inflammatory mediators detected in the serum and effluent in TBI patients compared to non-TBI patients could be associated with sustained inflammatory response stemming from the impact of central nervous system injury on post-injury inflammation.

We next sought to use DyHyp analysis to define potential drivers of cross-compartment spread of inflammation following combat injury. Over the interval n1-n3, TNF-α increased in the effluent in TBI patients but was remained constant in both the serum and effluent in non-TBI patients. In the next dynamic interval, n2-n4, TNF-α continued to rise in the wound effluent of TBI patients and began to rise in the serum. However, in non-TBI patients, no such rise in TNF-α was observed in either serum or effluent. This suggests the level of TNF-α may be associated with a sustained neural regulatory response not present in non-TBI patients. The rise in TNF-α in the serum in TBI patients over n2-n4 was followed by a rise in IL-6 and IL-17A in the serum over the interval n3-n5. No rise in IL-6 or IL-17A is observed at any time point in the serum or effluent in non-TBI patients (Fig. 6C–E). We further hypothesize that the consequent changes in IL-6 and IL-17A levels may also be associated with a disrupted neural control in attenuating Th17 cell responses.

Unlike patients with TBI, non-TBI patients experience a decline in IL-17A levels in the serum over the interval n2-n4. This fall in IL-17A in the serum occurs at the same time as a decrease in IFN-γ in both serum and wound effluent. Furthermore, the decrease in IFN-γ over n2-n4 and n3-n5 was associated with a reduction in number of significantly declining mediators over the same dynamic intervals, suggesting that IFN-γ-associated pathways may drive the attenuation of both local and systemic inflammation in the absence of TBI (Fig. 6C–E).

## Discussion

In the present study, we characterized dynamic networks of local and systemic inflammation in complex combat injures either with or without TBI. Our findings suggest the following novel findings:

- The presence of a robust, cross-compartment inflammatory response that is more complex in TBI than non-TBI combat casualties,
- A central role for IL-17A and Th17 responses in both the systemic circulation and local wounds of combat casualties with TBI, supporting a role for neural regulation of Th17 responses,
- The development of a novel Dynamic Hypergraph framework for assessing cross-tissue interactions over time,
- The suggestion, based on assessment of the dynamic evolution of inflammatory networks, that pro-inflammatory Th17 cells may efflux from the systemic circulation into the wound to counteract wound infection in trauma patients with TBI, and
- The inferred role of TNF-α in promoting—and IFN-γ in resolving—neurally regulated inflammatory responses involving IL-17A.

While a recent study that defined civilian trauma endotypes using a multi-omic approach pointed to IL-17A as one biomarker of a trauma endotype enriched for TBI^13^, this is the first study in humans specifically linking TBI in combat trauma to elevated wound and systemic IL-17A. As such, our study links injury-associated Th17 immune responses^13,17,21,24,46^ to the well-established negative impact of TBI on trauma outcomes^47–49^. Our studies may also connect the emerging role of IL-17A and Th17 immune responses in wound healing^50^ to the documented but not yet fully elucidated impact of systemic inflammatory responses on local inflammation in the wound^23^. That IL-17A was increased only in TBI patients when wound number was controlled for, suggests that IL-17A may be regulated in part by the central nervous system, as previously described for the chemokine IP-10/CXCL10^15^.

While the present study did not examine brain-localized inflammatory networks, our results are consistent with those of prior experimental studies involving TBI. The pro-inflammatory activity of IL-17A, which is produced by the IL-23/IL-17 axis, has also been associated with the pathogenesis of TBI in a rat model of secondary brain injury after TBI^51^. In another study, administration of taurine effectively mitigated the severity of brain damage by attenuating the increase of astrocyte activity and edema as well as pro-inflammatory cytokines including GM-CSF, IFN-γ, IL-17A, and TNF-α^52^. In a model of severe, penetrating TBI, intravenous delivery of simvastatin provided significant protection against injury-induced cognitive dysfunction and reduced TBI-specific serum levels of IL-17A^53^. More recently, propofol, a commonly used anesthetic, alleviated brain injury in rats with TBI and maintained the Th17/Treg balance^54^. Notably, IL-17A derived predominantly from microglia has also been implicated in the pathobiology of various neurodegenerative diseases such as Alzheimer’s and Parkinson’s’ disease^55^. Further studies are needed to determine if there is a connection between IL-17A production in wounds, the systemic circulation, and the brain following trauma with TBI.

Th 17 cells have been divided broadly into two sub-groups: nonpathogenic Th17 cells that express both IL-17A and IL-10 and highly inflammatory, pathogenic Th17 cells that upregulate GM-CSF and down-regulate the host-protective IL-10^56,57^. In our study comparing time-courses and dynamic networks of inflammation in the systemic circulation of military patients, we found a higher overall network complexity and mediator connectivity, including IL-17A, in serum of TBI patients as compared to non-TBI casualties. We have suggested previously that trends toward increasing inflammatory network complexity as assessed by DyNA are often associated with adverse outcomes such as mortality following trauma^17,18,21^. In the present study, the elevated systemic levels and positive correlation of IL-17A with GM-CSF, not with IL-10, supports the hypothesis of a type 17 immune response skewed towards a pro-inflammatory state characterized—and possibly driven by—pathogenic Th17 cells in trauma patients with TBI. Further studies with a larger panel of mediators are needed to define the potential beneficial effect of targeting IL-17A in the setting of TBI.

Acute inflammation is mediated by local, hormonal, and neuronal mechanisms, all of which rely of TNF-α as a key, feed-forward inflammatory mediator^59^. Our DyHyp analyses suggest that TBI results in a greater pro-inflammatory response that begins with the expression of TNF-α in the effluent during the first dynamic interval and then the appearance of TNF-α in both the serum and effluent in the second dynamic interval. Consistent with the known role of neural pathways in regulating cytokines such as TNF-α and IL-6^60^, TNF-α was not significantly upregulated in non-TBI patients. This finding supports the hypothesis that neural regulation is necessary to prevent the excessive significant rise in TNF-α and concomitant rise in IL-6 and IL-17A. Interferon-γ, a conventionally pro-inflammatory molecule often present in neurologic inflammatory diseases, is now more appropriately considered a modulator of both pro- and anti- inflammatory responses^61^. In line with recent discoveries of its anti-inflammatory properties, IFN-γ was inferred to be decreasing in the serum and effluent in non-TBI patients and increasing in the serum in TBI patients during the intervals n2-n4 and n3-n5. One proposed explanation for this phenomenon stems from knowledge that brain astrocytes produce TNF-α, IL-6, and other inflammatory mediators in response to IFN-γ. In the absence of CNS inflammation in non-TBI patients, IFN-γ decreased over time. This reduction in IFN-γ could plausibly attenuate the astrocyte-mediated secretion of TNF-α and IL-6, thereby further reducing IL-17A-mediated inflammatory responses in the serum and effluent^62,63^. Furthermore, we hypothesize that microglia-mediated neuroprotective effects are reduced in response to IFN-γ stimulation^64^. We further hypothesize that protective, neurally mediated effects of IFN-γ may prevent upregulation of inflammatory mediators in patients with combat wounds.

To discern whether the computational analyses suggesting that TBI is not merely associated with more complex inflammation, we rely on past analyses of TBI and wound healing domains. Compared to non-survivors of TBI, survivors of TBI manifested differential roles for IL-6, one of which included propagating TNF-$\alpha$ mediated inflammation^65^. Non-survivors of TBI also exhibited elevated TNF-$\alpha$ that was mediated by both IL-6 and IL-8^65^. Our DyHyp analysis suggests that TNF-$\alpha$ serves as a cross-compartmental bridge that facilitates an upregulation in IL-8 and IL-6 in the serum, even when IL-6 is being attenuated in local injuries. The inferred neural regulation of TNF-$\alpha$ suggests at least one mechanism for propagating inflammatory responses in injured patients with TBI.

Many of the insights, inferences, and hypotheses discussed above were facilitated by a workflow involving multiplexed assessment of immune/inflammatory mediators coupled with dynamic network inference, and specifically DyHyp. We posit that while tools such as DyNA and Dynamic Bayesian Network (DyBN) inference have been of tremendous value in elucidating novel aspects of trauma-induced inflammation^10,66^, tools such as hypergraphs^24^ and now DyHyp are necessary to explicitly infer dynamic inflammatory processes across tissue compartments. This is especially so when attempting to infer the role for a compartment, such as the central nervous system, that is not assessed explicitly.

Our studies must be interpreted in the context of several limitations. These limitations include some heterogeneity of sampling given the sample acquisition at debridement intervals rather than standard time points; the heterogenous number of wounds in individual patients; the limited number of analytes (especially with regard to wound infection) that could be assessed; and the absence of an independent validation cohort of combat casualties. Computationally, tools such as mechanistic mathematical models^11,12^ might yield more precise and testable insights into the inflammatory responses of these patients. Additionally, our study cohorts were predominantly male. While a recent study in rodents found no sex-based differences in various pathological features of TBI including splenocyte cell proliferation, activation of microglia, and inflammatory cytokine production at 6 h post-injury^58^, future multi-center studies in diverse cohorts of TBI patients remain necessary to confirm this equivalence. We also note that, in addition to TBI, psychiatric and neurological factors (e.g., PTSD) can have an impact on the systemic stress response and need to be considered in future studies. Finally, adding an extensive panel of multi-omic analytes assessed in both wounds and the systemic circulation will likely add substantial new knowledge.

In conclusion, despite a heterogeneous inflammatory response in individual patients, our studies suggest that a network-based analysis may have the potential to clearly identify TBI-based inflammatory responses in trauma patients. Furthermore, these combined clinical and computational studies suggest a novel role for Th17 immune responses following combat trauma and TBI, which may serve to counteract wound infection at the cost of elevated and sustained systemic inflammation and attendant sequalae. Ultimately, we suggest that combined approaches using network-based analysis of biomarkers and related machine learning approaches may help better identify possible treatment complications and aid in the diagnosis and management of both combat casualties and civilian polytrauma victims.

## Supplementary Information

Supplementary Information.

## Data Availability

The datasets generated during and/or analyzed during the current study are available from the corresponding author on reasonable request.
