# Supplementary material for: Central role for neurally dysregulated IL-17A in dynamic networks of systemic and local inflammation in combat casualties
Source: Sci Rep. 2023 Apr 24;13:6618. doi: 10.1038/s41598-023-33623-z (PMC10126120; doi:10.1038/s41598-023-33623-z)
Supplement: Supplementary file 1 — Supplementary Information. [file 41598_2023_33623_MOESM1_ESM.pdf]

Zamora et al.

Central Role for Neurally Dysregulated IL-17A in Dynamic Networks of Systemic and Local Inflammation in Combat Casualties

Supplementary Material

Suppl. Fig. 1. Time-dependent release of inflammatory mediators in serum samples of TBI vs. non-TBI patients. Serum samples from TBI (n=59) and non-TBI (n=81) patients were obtained at five consecutive debridements and assayed for 23 inflammatory/immune mediators using a human multiplex Luminex™ assay as described in *Materials and Methods*. Concentrations are expressed in pg/ml + SEM and comparison was performed by Two-Way ANOVA as described in *Materials and Methods*.

Suppl. Fig. 1

Serum Inflammatory Mediators

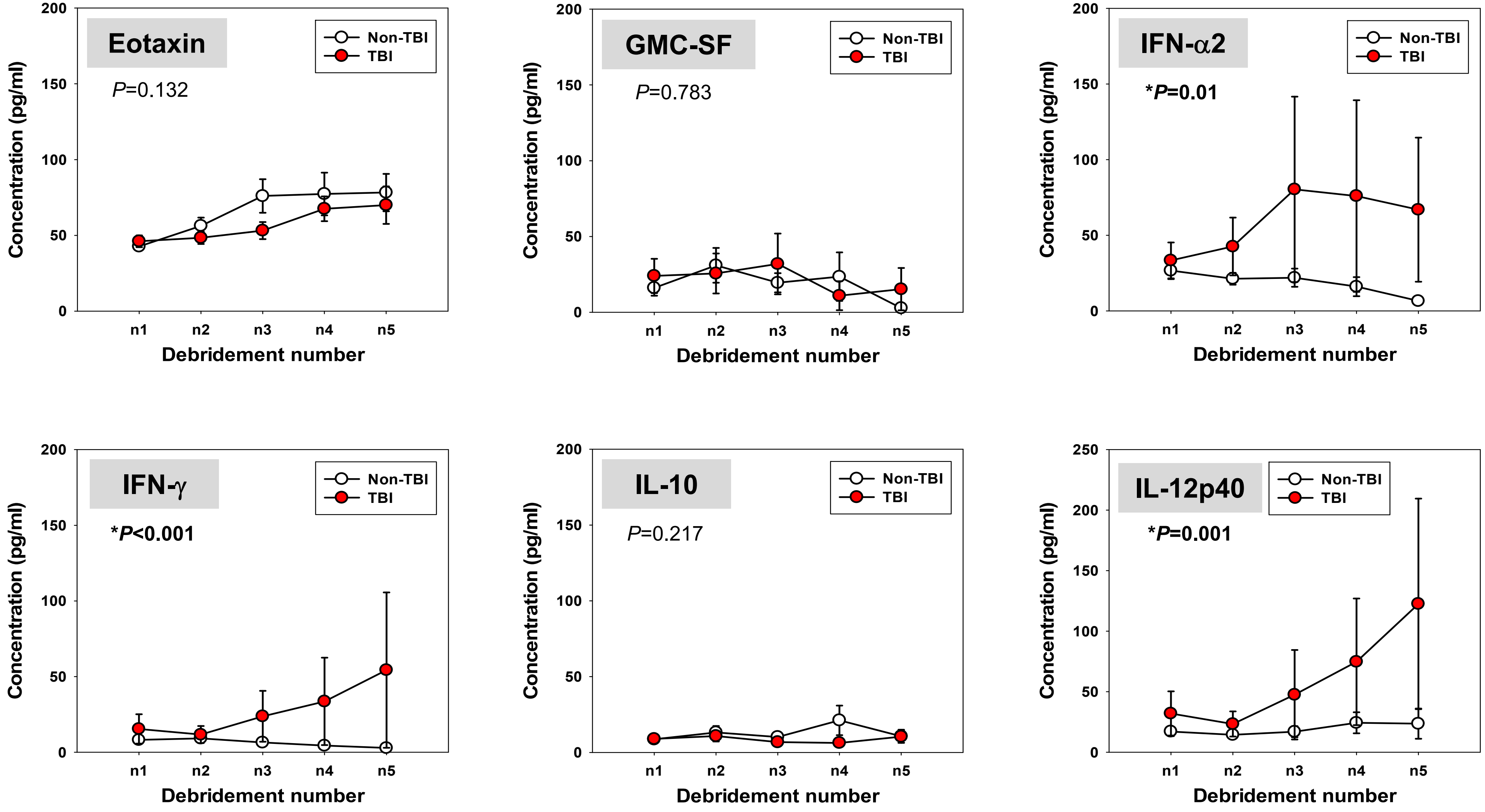

Suppl. Fig. 1

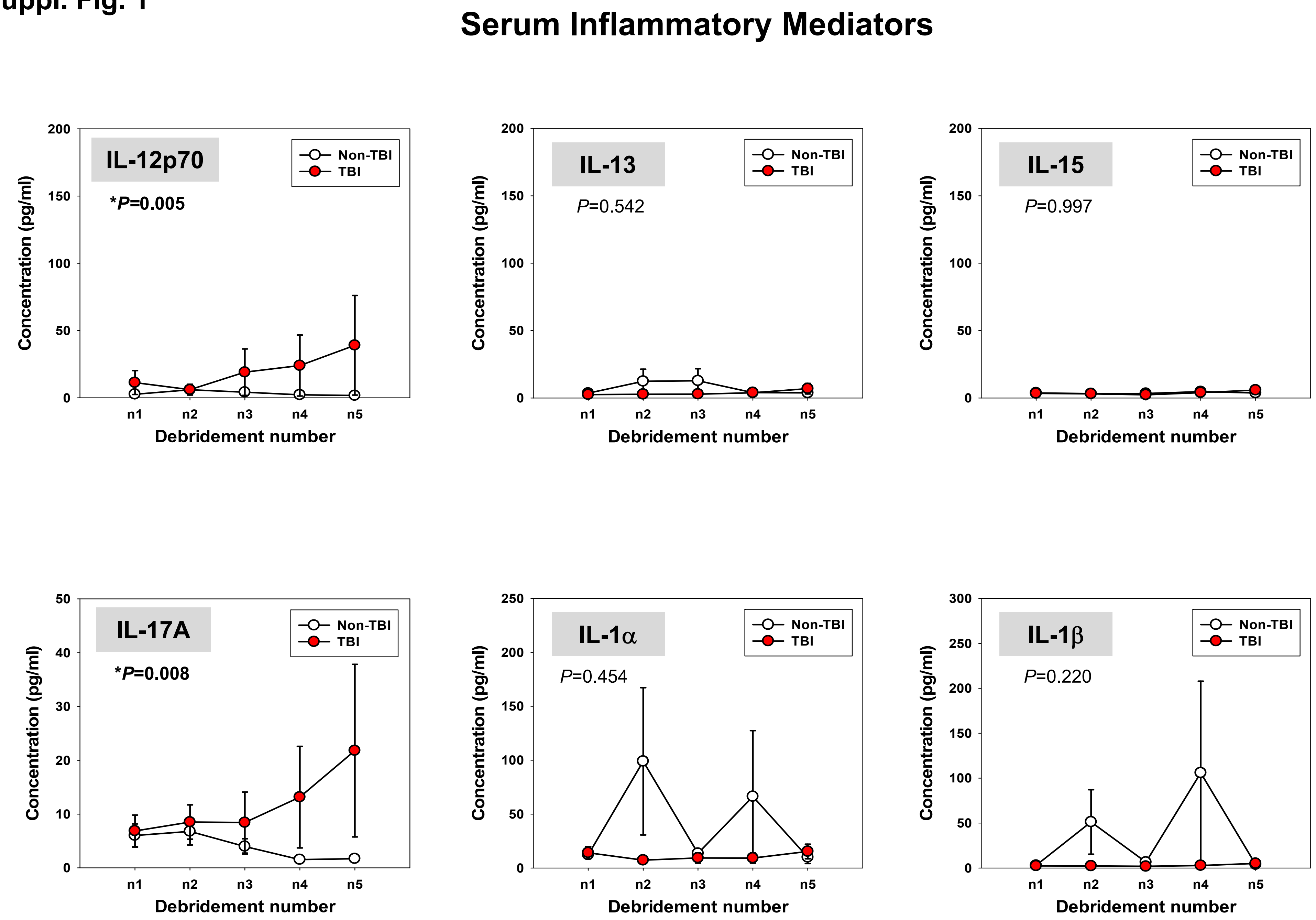
Serum Inflammatory Mediators
IL-12p70
*P=0.005
IL-13
P=0.542
IL-15
P=0.997
IL-17A
*P=0.008
IL-1α
P=0.454
IL-1β
P=0.220
Non-TBI
TBI
Concentration (pg/ml)
Debridement number
n1
n2
n3
n4
n5

Suppl. Fig. 1

Serum Inflammatory Mediators

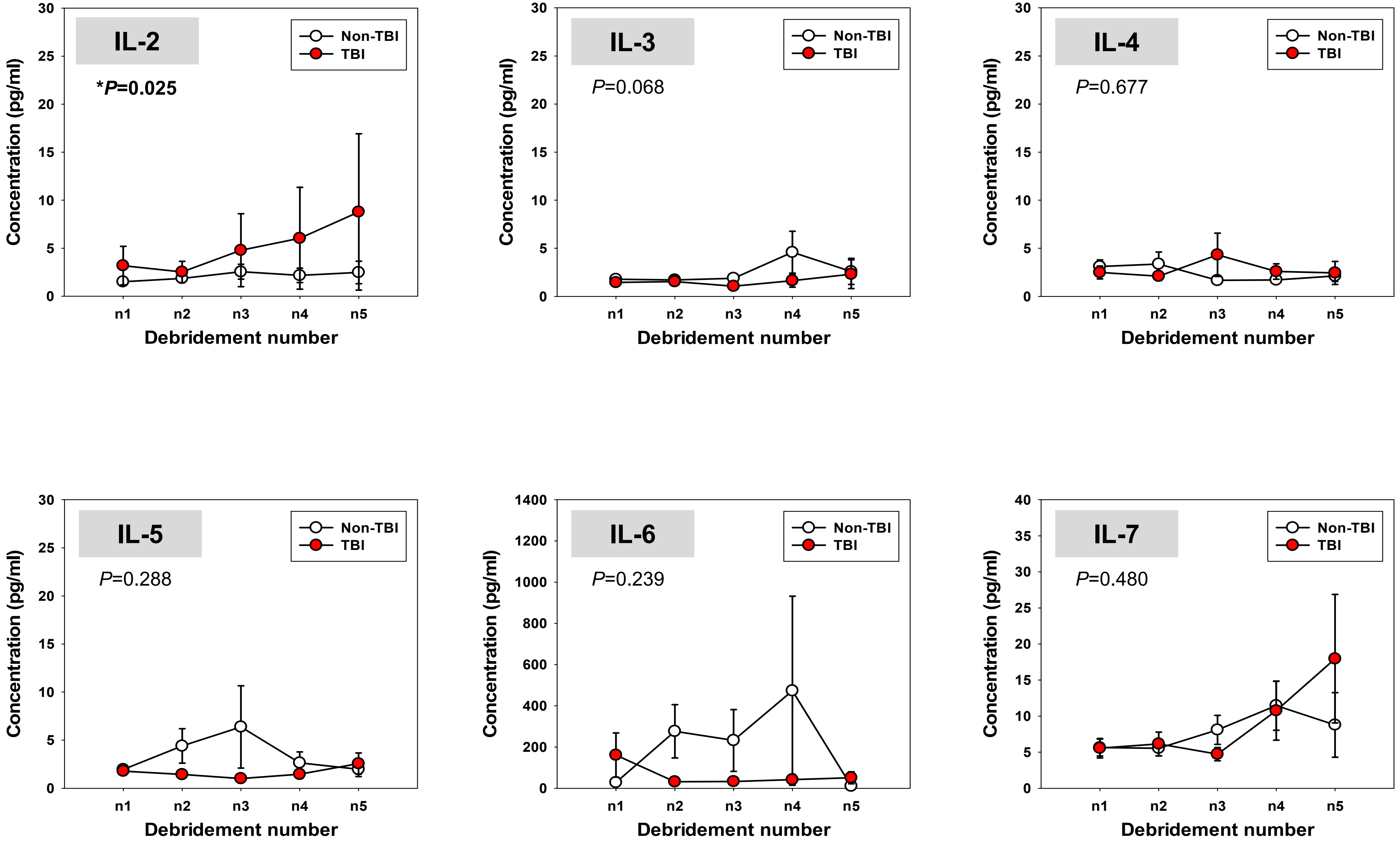

Suppl. Fig. 1

Serum Inflammatory Mediators

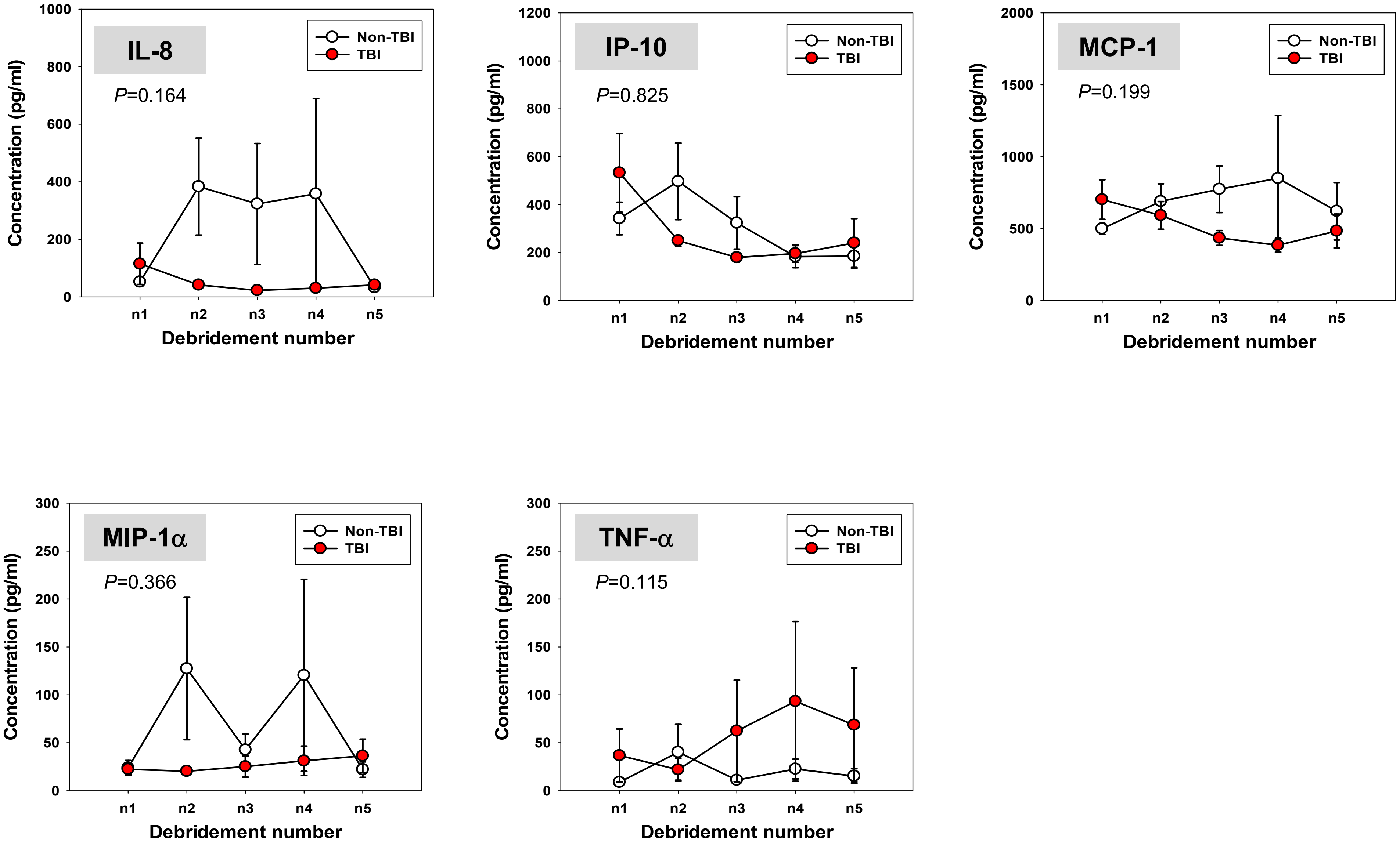

Suppl. Fig. 2. Time-dependent release of inflammatory mediators in wound effluent samples of TBI vs. non-TBI patients. Wound effluent samples from TBI (n=59) and non-TBI (n=81) patients were obtained at five consecutive debridements and assayed for 23 inflammatory/immune mediators using a human multiplex Luminex™ assay as described in *Materials and Methods*. Concentrations are expressed in pg/ml + SEM and comparison was performed by Two-Way ANOVA as described in *Materials and Methods*.

Suppl. Fig. 2

Effluent Inflammatory Mediators

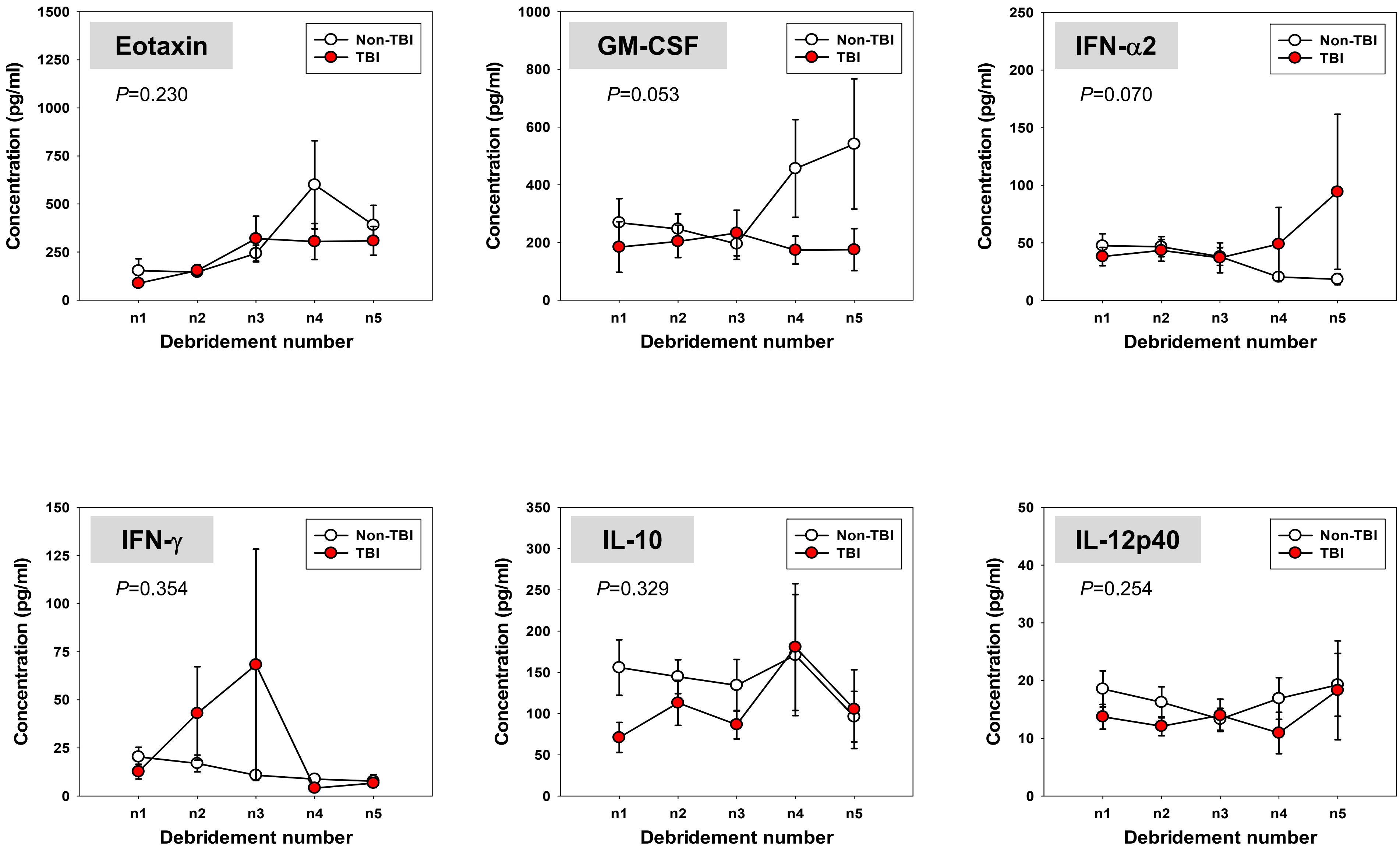

Suppl. Fig. 2

Effluent Inflammatory Mediators

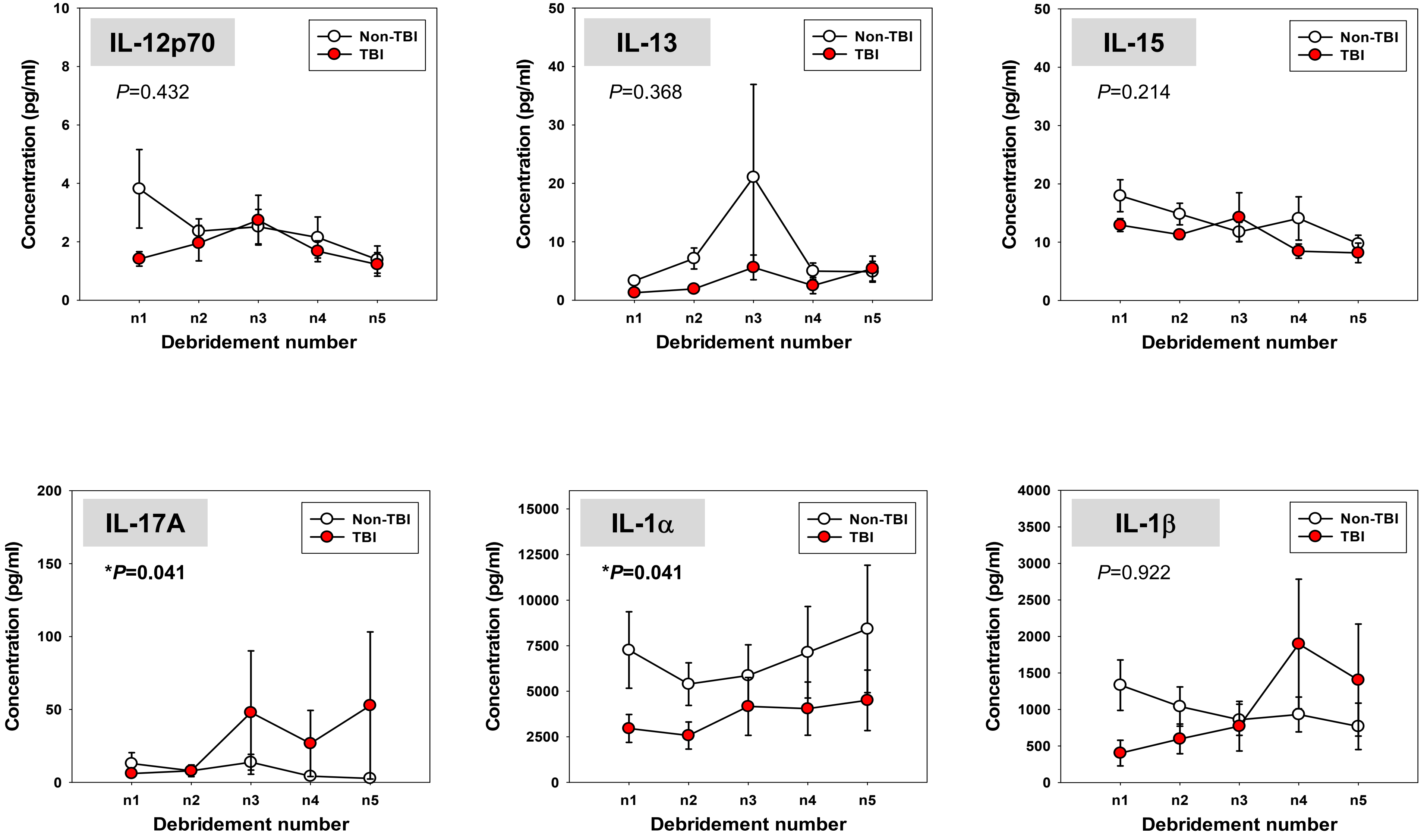

Suppl. Fig. 2

Effluent Inflammatory Mediators

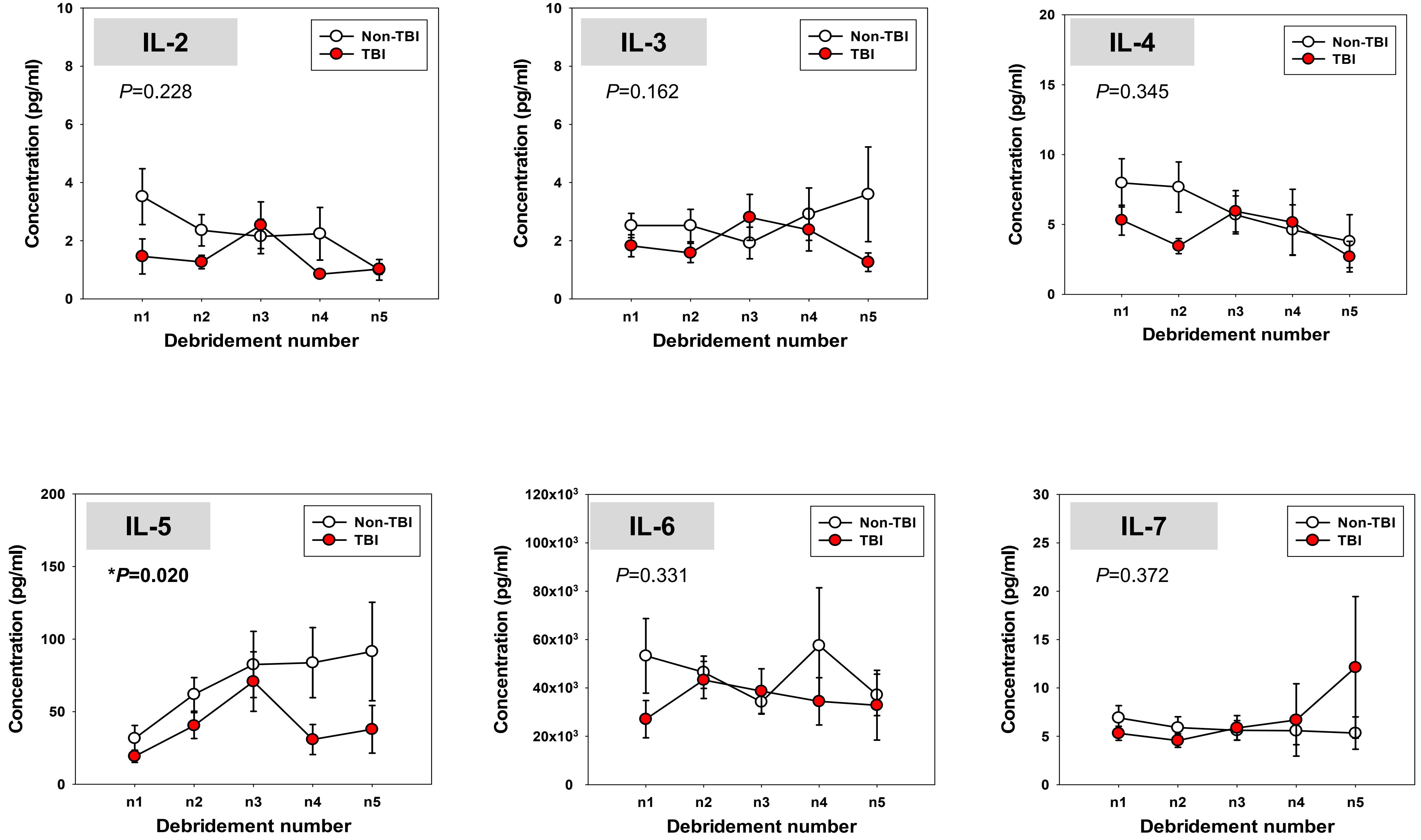

Suppl. Fig. 2

Effluent Inflammatory Mediators

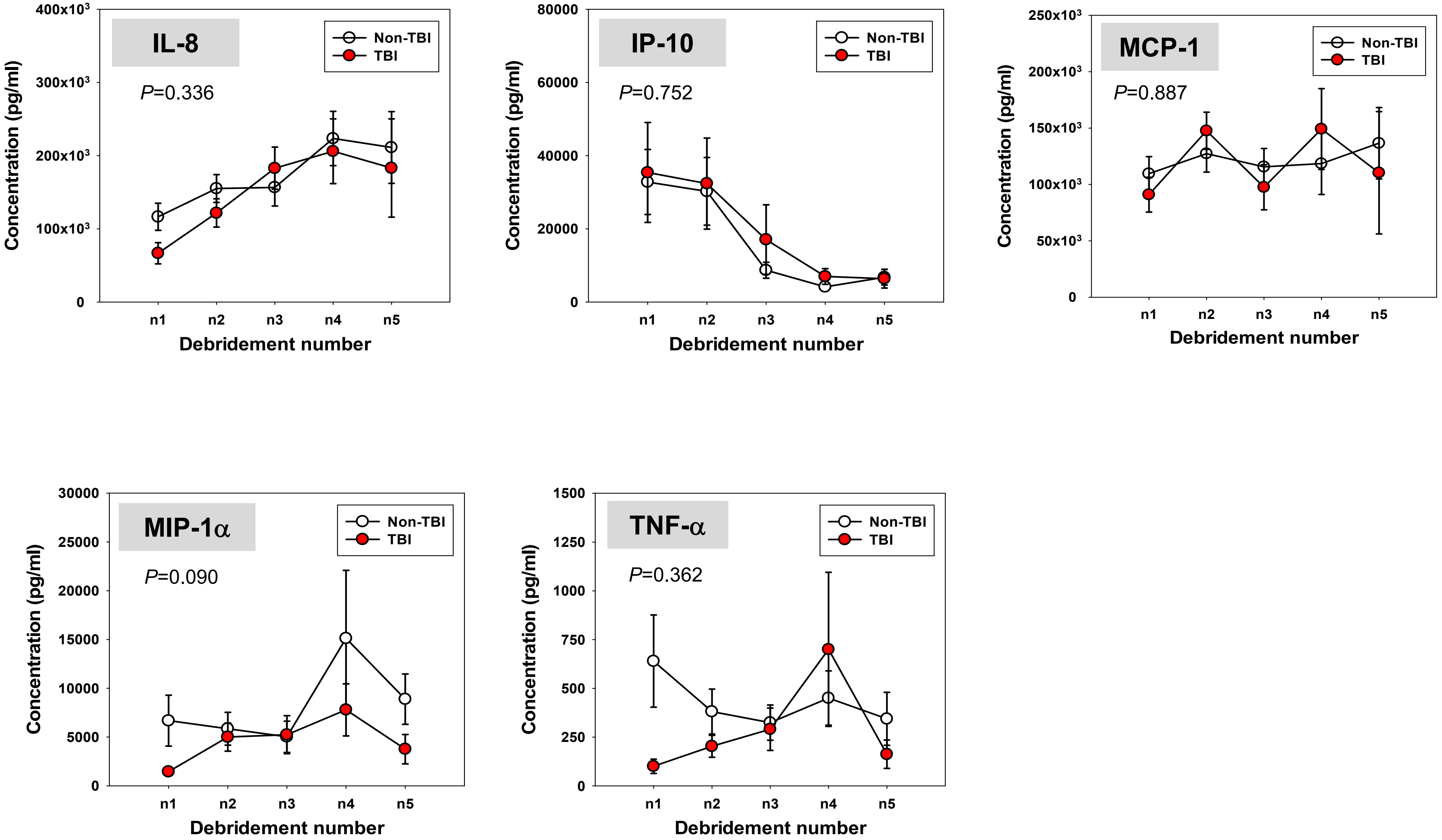

Suppl. Fig. 3. Dynamic inflammatory networks in TBI vs. non-TBI trauma patients. Circulating inflammatory mediators in serum and effluent samples from TBI and non-TBI patients who sustained injuries due to Blast only (see Table 2) were measured, and DyNA (stringency level = 0.95) was performed during each of four debridement intervals (n1-n2, n2-n3, n3-n4, n4-n5) using MATLAB® software as described in *Materials and Methods*. Panels **A** and **B** show an overview of all the networks and mediator connections in both patient subgroups in (A) serum and (B) effluent. Panels **C** (serum) and **D** (effluent) show the network complexity for each patient subgroup during each of the four debridement intervals calculated as described in *Materials and Methods.*

Suppl. Fig. 3

Patients (Blast injury)

DyNA

A

Serum

n1-n2 | n2-n3 | n3-n4 | n4-n5

Non-TBI

TBI

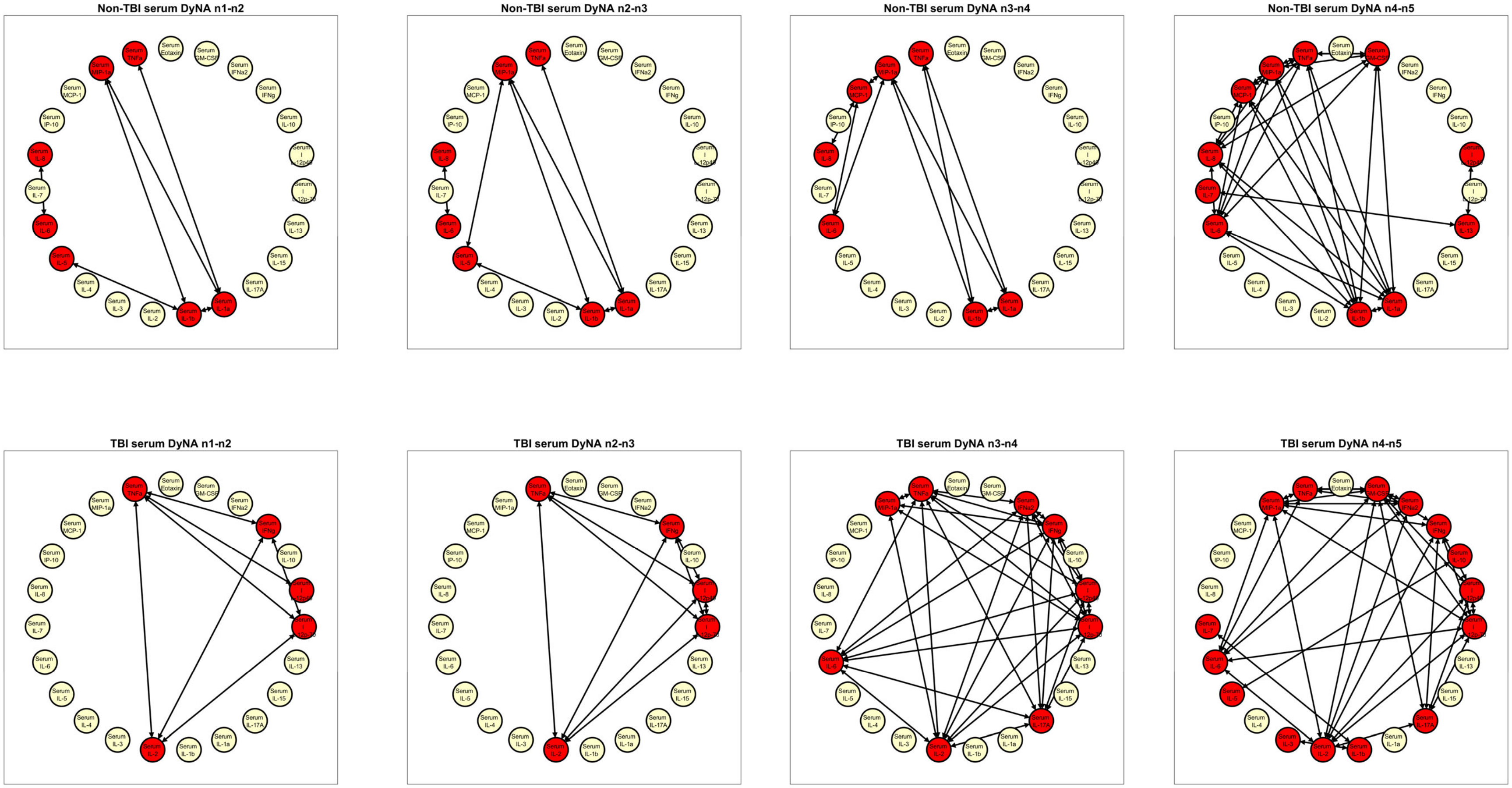

stringency level 0.95

Suppl. Fig. 3

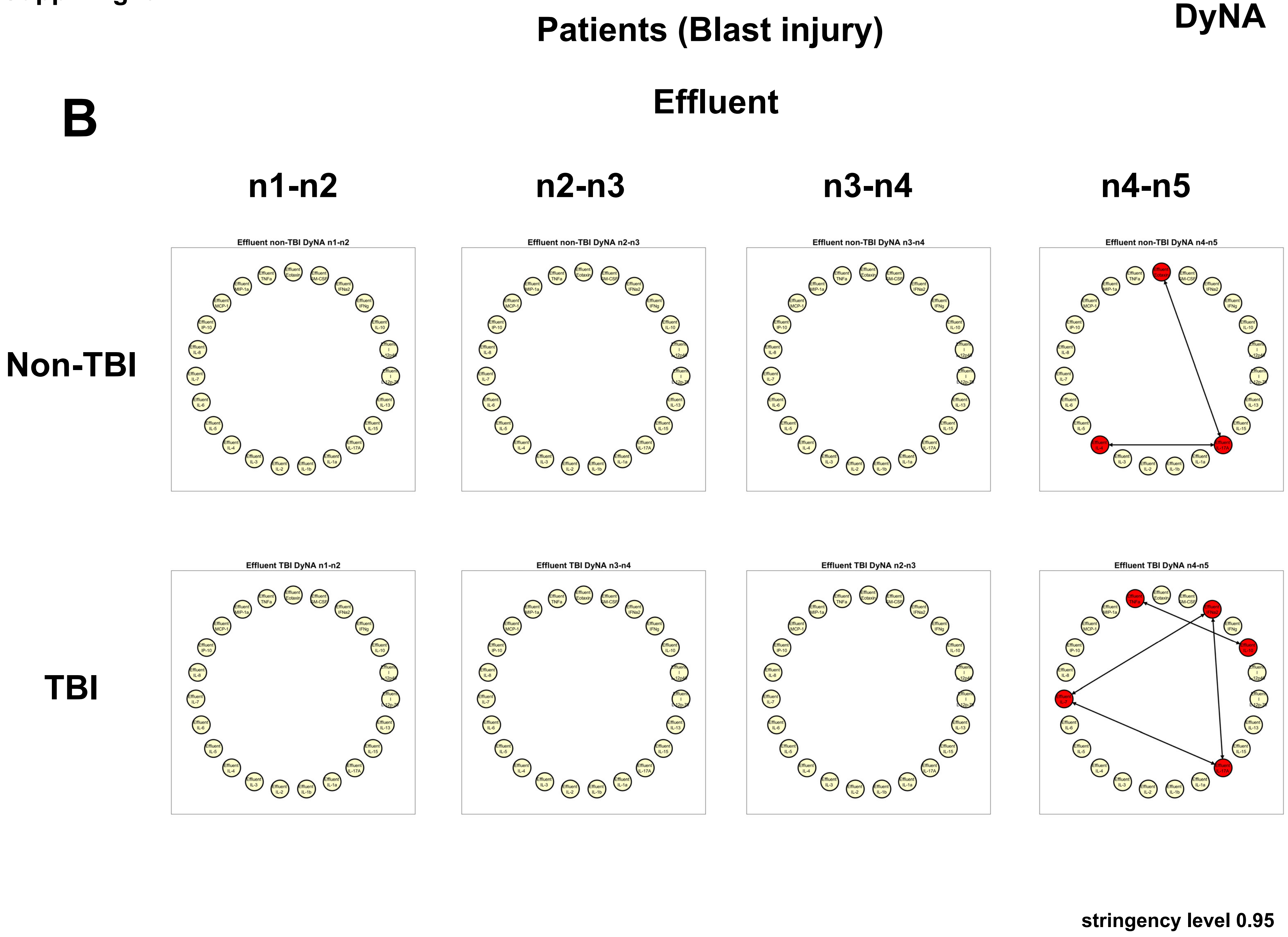

Patients (Blast injury)
DyNA
B
Effluent
n1-n2
n2-n3
n3-n4
n4-n5
Non-TBI
TBI
Effluent non-TBI DyNA n1-n2
Effluent non-TBI DyNA n2-n3
Effluent non-TBI DyNA n3-n4
Effluent non-TBI DyNA n4-n5
Effluent TBI DyNA n1-n2
Effluent TBI DyNA n3-n4
Effluent TBI DyNA n2-n3
Effluent TBI DyNA n4-n5
stringency level 0.95

Suppl. Fig. 3

Patients (Blast injury)

DyNA

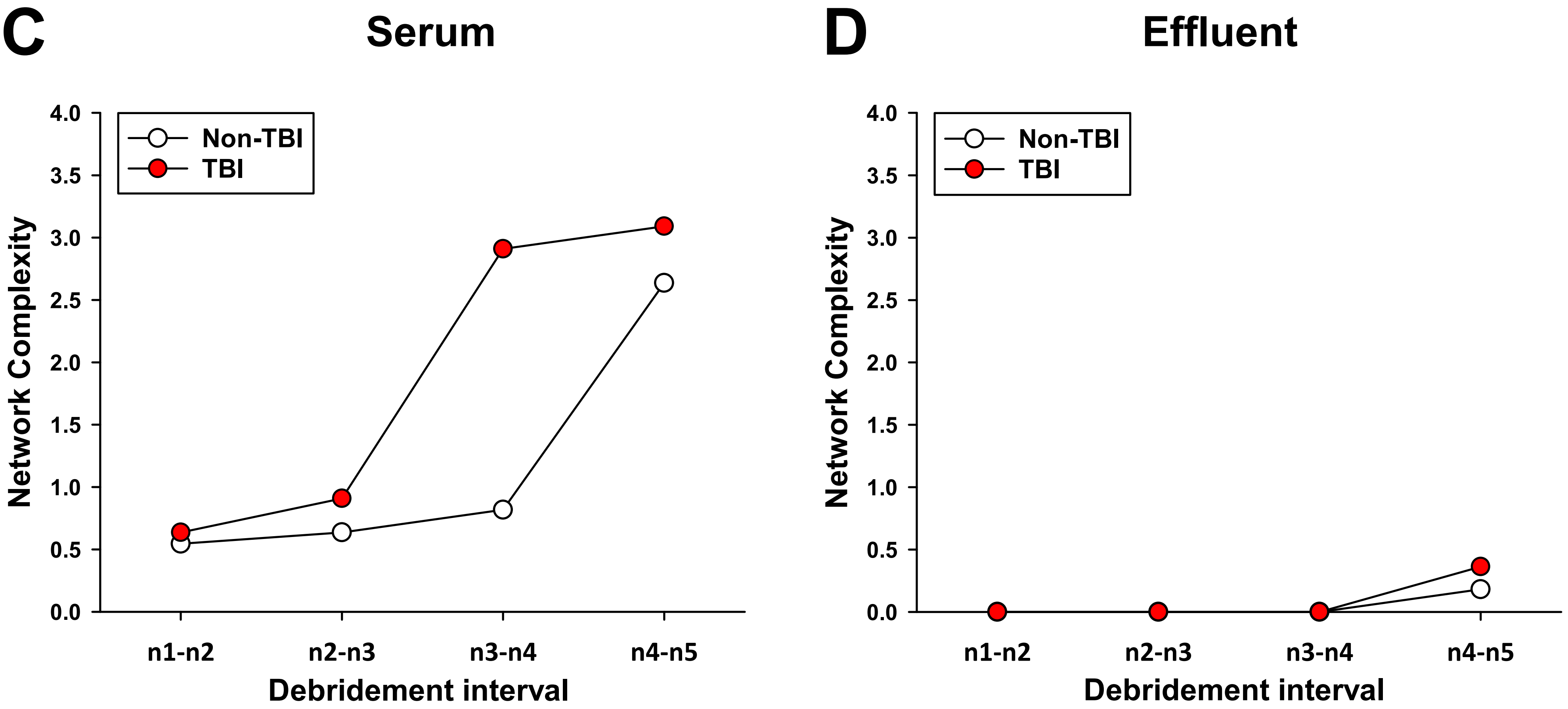

stringency level 0.95

Suppl. Table 1

Number of connections/mediator and debridement interval in inflammatory networks of non-TBI and TBI patients (see Fig. 2A [serum] and Fig. 2B [effluent])

Patient group	Debridement	IFNa2	IFNg	IL-12p40	IL-12p70	IL-17A	IL-2	IL-1a	IL-5
Serum Non-TBI-1	n1-n2	0	0	0	0	0	0	3	0
Serum Non-TBI-2	n2-n3	0	0	0	0	0	0	3	0
Serum Non-TBI-3	n3-n4	0	0	0	0	0	0	1	0
Serum Non-TBI-4	n4-n5	0	0	1	0	0	0	6	0
Serum TBI-1	n1-n2	0	3	1	3	0	3	0	0
Serum TBI-2	n2-n3	0	4	4	4	0	4	0	0
Serum TBI-3	n3-n4	7	8	7	8	7	8	0	0
Serum TBI-4	n4-n5	5	6	5	7	5	8	0	1

Patient group	Debridement	IFNa2	IFNg	IL-12p40	IL-12p70	IL-17A	IL-2	IL-1a	IL-5
Effluent Non-TBI-1	n1-n2	0	0	0	0	0	0	0	0
Effluent Non-TBI-2	n2-n3	0	0	0	0	0	0	0	0
Effluent Non-TBI-3	n3-n4	0	0	0	0	0	0	0	0
Effluent Non-TBI-4	n4-n5	0	0	0	0	1	0	0	0
Effluent TBI-1	n1-n2	0	0	0	0	0	0	0	0
Effluent TBI-2	n2-n3	0	0	0	0	0	0	0	0
Effluent TBI-3	n3-n4	0	0	0	0	0	0	0	0
Effluent TBI-4	n4-n5	2	0	0	0	2	0	0	0

Mediators with significant changes (TBI vs. non-TBI) analyzed by Two-Way ANOVA (see Suppl. Figures 1 and 2)

Serum :	IFNa2, IFNg, IL-12p40, IL-12p70, IL-17A, IL-2
Effluent :	IL-17A, IL-1a, IL-5
Serum and Effluent :	IL-17A
